# Vedolizumab Dose Escalation Resolves Immune Checkpoint Inhibitor–Induced Colitis in Two Patients

**DOI:** 10.1155/crom/7637337

**Published:** 2025-09-23

**Authors:** Cong Wang, Nikhil Nayee, Jacqueline M. Garrick, Justin Moser

**Affiliations:** ^1^College of Medicine, University of Arizona, Phoenix, Arizona, USA; ^2^Department of Medical Oncology, HonorHealth Research Institute, Scottsdale, Arizona, USA

**Keywords:** case report, case series, colitis, dose escalation, immune checkpoint inhibitor, vedolizumab

## Abstract

Colitis is a common side effect of all currently approved immune checkpoint inhibitors (ICIs). Vedolizumab is an *α*_4_*β*_7_ monoclonal antibody approved for treating inflammatory bowel disease (IBD) and is commonly used off-label to manage checkpoint inhibitor colitis (CIC). Previous studies have attempted dose escalation of vedolizumab for clinically unresponsive IBD patients and have been able to achieve clinical remission in 49.6% of these patients without an associated increase in adverse events. However, this has yet to be reported in patients with CIC. Here, we report on two patients who developed CIC after being treated with ICIs for metastatic cancer and received a double dose of vedolizumab. Both patients' colitis was initially treated with infliximab, steroids, and standard 300-mg vedolizumab dosage with an incomplete response. When administered a double dose of 600-mg vedolizumab, colitis symptoms were resolved without further recurrence or the need for treatment in both patients. This case series outlines effective clinical experience in treating patients with CIC refractory to standard vedolizumab dosage and supports further study of vedolizumab dose escalation in this patient population.

## 1. Introduction

Immune checkpoint inhibitor (ICI)–induced colitis is a common side effect of all approved ICIs. The risk of checkpoint inhibitor colitis (CIC) ranges from 9.2% to 44.1%, depending on the specific ICI and combination therapy used [1, 2]. Since ICIs function by blocking coinhibitory receptors, such as cytotoxic T lymphocyte–associated protein 4 (CTLA-4), programmed death protein 1 (PD-1), or programmed death-ligand 1 (PD-L1), and promoting immune-mediated tumoral cell death, their use can result in immune system overactivity and humoral autoimmunity [3–6]. While specific mechanisms of CIC are not fully understood, a study by Terrin et al. [5] formulated three categories of mechanisms: (1) on-target effects, referring to antitumor activity that also affects normal tissue, (2) off-target effects, the upregulation of inflammatory cytokine pathways such as tumor necrosis factor alpha (TNF-*α*) and interleukin 17 (IL-17), and (3) host-related factors, such as the bowel microbiome diversity. Regarding the on-target effects, increased infiltration of CD4+ T cells into the bowel has been found in subjects treated with anti-CTLA-4 ICIs, while increased infiltration of CD8+ cells into the bowel has been seen in subjects treated with anti-PD-1 ICIs. In both cases, these increases are correlated with increased severity of CIC [5, 7–9].

Vedolizumab is an FDA-approved *α*_4_*β*_7_ monoclonal antibody that induces and maintains remission in patients with moderate to severe inflammatory bowel disease (IBD) [10, 11]. Given its lack of systemic immunosuppression, it is commonly used to manage CIC [12]. The *α*_4_*β*_7_ receptor, which is present on memory/effector T cells, normally binds to mucosal addressin cellular adhesion molecule (MAdCAM-1) on Peyer's patches and other intestinal lymphoid tissues to draw T cells to the intestine [13]. Studies suggest that vedolizumab acts by selectively inhibiting the migration of specific T cell subtypes [13]. The current standard dosing regimen for vedolizumab in IBD includes intravenous (IV) induction dosing administered as a 300-mg flat dose over 30 min at Weeks 0, 2, and 6, followed by maintenance infusions every 8 weeks thereafter [11]. For CIC, vedolizumab dosing is variable, ranging from a single 300-mg dose to the standard induction and maintenance regimen for IBD, although maintenance therapy is commonly not needed.

Vedolizumab exhibits a dose–response relationship with clinical outcomes, with IBD patients achieving higher trough levels having a greater likelihood of continuing treatment at 1 year due to ongoing clinical benefit [14]. Early trough levels above 16.55 *μ*g/mL appear closely associated with treatment persistence over the first year and with histological and mucosal healing [14–16]. Multiple studies have intensified vedolizumab dosing for clinically unresponsive IBD patients by administering it every 4 weeks instead of the standard 8 weeks during maintenance. This approach has achieved clinical remission in 49.6% of patients, with remission rates ranging from 40.0% to 73.3% across studies [17–23]. Despite escalation, there were no reported increases in adverse risks, although hives and pruritus were reported in two patients [17–23].

Dose escalation by double dosing from 5 to 10 mg/kg for TNF-*α* inhibitors is common practice in the treatment of IBD for patients who are hypoalbuminemic or do not have an initial response to therapy. In one study, clinical benefit was regained in 72.4% of infliximab-treated patients and 52.3% of adalimumab-treated patients after increasing the dose to 10 mg/kg [24]. However, there is limited data on dose doubling for hypoalbuminemic patients on newer IBD medications, such as vedolizumab.

In this study, we report on two cases of CIC: a 68-year-old woman with melanoma and a 53-year-old woman with colon cancer, both refractory to standard therapy and subsequently treated with a flat dose of 600-mg vedolizumab, double the standard dose of 300 mg.

## 2. Case Presentation

### 2.1. Case #1

A 68-year-old woman with refractory metastatic melanoma was treated with a CTLA-4 inhibitor after the progression of her disease on frontline therapy. A month into treatment, she developed CIC and was treated with 5 mg/kg of infliximab IV and 1 mg/kg of methylprednisolone daily. This regimen resolved her diarrhea until the next month, at which time her CIC relapsed while the patient was on a standard prednisone taper, and she began to have up to six bowel movements per day. For comparison, her baseline bowel patterns before relapse were 1–2 stools a day. To address this, she was continued on methylprednisolone and received a second dose of infliximab. Over the next 2 weeks, she had a brief improvement in the number of loose stools but eventually developed severe diarrhea of up to 10 episodes per day. Subsequently, her treatment was changed to vedolizumab 300 mg IV. She was treated with the standard Week 0 and 2 induction doses but again had worsening CIC by the time of her Week 6 dose. Given her body habitus and the flat dosing of vedolizumab, subtherapeutic trough levels were suspected. However, an assessment determined that waiting for testing of trough levels was not in the patient's best interest due to the slow turnaround time of such testing and the severity of her symptoms. Thus, she was given 600 mg of vedolizumab at Week 6. Improvement in her bowel symptoms began a few days after treatment with no recurrence of her colitis, and no adverse reactions were documented. No further treatment for CIC was required thereafter, even after the patient was tapered off steroids. CBCs were drawn at the time of the initial dose of 300-mg vedolizumab, at the time of the double dose of 600-mg vedolizumab, and 14 days post-double dose of vedolizumab; results are reported in Table 1.

### 2.2. Case #2

A 53-year-old woman with metastatic colon cancer previously treated with chemotherapy and surgical resection was treated with CTLA-4-based combination ICI therapy. Two months after starting ICI therapy, she began to have diarrhea and was started on 1 mg/kg of methylprednisolone daily and 5 mg/kg of infliximab. Although she did have partial improvement in her symptoms, her diarrhea did not fully resolve and eventually led to an ER visit. Her CIC treatment was then changed to vedolizumab 300 mg, which was planned to be administered via a standard three-dose induction series. At the time of her first vedolizumab dose, she was hypoalbuminemic with serum albumin of 1.5, a known correlate for inferior serum levels of monoclonal antibodies, most commonly TNF-*α* inhibitors, used in the treatment of IBD [25]. She had no improvement in symptoms after this first dose, and her albumin continued to drop. Therefore, she received a double dose of 600-mg vedolizumab for her 2-week induction dose, which would have been a common practice for other CIC therapies like infliximab. Over the next few days, her colitis symptoms and diarrhea gradually improved and eventually resolved. No adverse reactions were documented following the double dose of vedolizumab. She was eventually tapered off prednisone and required no further CIC therapy. CBCs were drawn at the time of the initial dose of 300-mg vedolizumab, at the time of the double dose of 600-mg vedolizumab, and 1 month post-double dose of vedolizumab; results are reported in Table 1.

## 3. Discussion

In the present study, we describe two cases of CIC in which patients had an incomplete response to standard 300-mg vedolizumab dosing and were therefore given a double dose of 600-mg vedolizumab, resulting in the complete resolution of colitis symptoms shortly thereafter. Vedolizumab treatment for CIC aligns with the current guidelines from the American Society of Clinical Oncology (ASCO) for cases refractory to initial corticosteroids, which recommend subsequent treatment with immunosuppressant biologics, such as infliximab, rituximab, or vedolizumab, or nonbiologic immunosuppressant agents, including cyclophosphamide or mycophenolate [26, 27]. While ASCO guidelines recommend the standard 300-mg vedolizumab dose administered per the approved label for IBD, clinical studies on optimal dosing strategies for CIC remain limited. Case studies, such as those presented here, offer valuable clinical insight to support more rigorous research on dosing strategies of vedolizumab for CIC and can help shape existing and future clinical practice.

The rationale for increasing the dosage of vedolizumab is supported by previous studies, which have observed a correlation between vedolizumab trough levels and clinical response in patients with IBD [14]. Early vedolizumab trough levels higher than 16.55 *μ*g/mL have been associated with treatment persistence over the first year and mucosal healing [14–16]. Unfortunately, although endoscopy before and after treatment would have been valuable data in these cases, due to current ASCO guidelines for the management of ICI side effects not recommending endoscopy for all cases of CIC, no assessment of mucosal healing can be made in either of the two cases presented here, as neither had colonoscopy before or after the initiation of the double dose of vedolizumab. In cases of IBD patients with a clinical lack of response to standard vedolizumab dosing, previous studies that have attempted dose escalation have achieved clinical remission in 49.6% of patients, with clinical remission rates varying from 40.0% to 73.3% across different studies [17–23]. However, some studies contradict this, instead suggesting that trough levels are not associated with clinical response [28, 29].

Despite the standard flat dosing regimen of vedolizumab, evidence suggests that patient weight may correlate with trough levels, with lower body mass being an independent predictor of higher vedolizumab trough levels and better mucosal healing in IBD patients [30, 31]. However, one study comparing dose escalation frequency and remission rates in obese and nonobese IBD patients found no statistically significant differences with patient weight [32]. Albumin levels may be another point of interest, as early evidence suggests that hypoalbuminemia impacts trough levels, with lower albumin levels associated with lower vedolizumab trough levels and mucosal healing [31]. One hypothesized link for this association is the neonatal Fc receptor (FcRn), which facilitates the salvage of albumin and IgG. This receptor has been found to be decreased in patients with conditions similar to CIC, such as ulcerative colitis [25]. Notably, hypoalbuminemia is a known adverse event associated with ICI therapy that correlates with poor prognosis [33]. Patients with CIC may experience a higher incidence of hypoalbuminemia compared to those with IBD, though further investigation is warranted to confirm this. Consequently, extrapolating the standard IBD dosing regimen to CIC patients may be inadequate, given the potential for fundamental differences in the pharmacokinetics of vedolizumab stemming from ICI therapy. Although these studies offer supportive evidence for individualized vedolizumab dose optimization, randomized, controlled trials are needed to assess the clinical and cost-effectiveness of this practice.

Limitations of this study include the lack of CTCAE grading of colitis and quantitative measures of CIC improvement. Although CBC values were obtained at each visit, the only consistent trend observed in both patients was increasing thrombocytosis, which cannot be solely attributed to the double dose of vedolizumab due to the nature of this study. Additionally, we did not measure inflammatory markers. While ASCO and the Society for Immunotherapy of Cancer (SITC) guidelines for the management of immune-related adverse events, such as CIC, recommend measuring fecal calprotectin based on institutional availability, the turnaround time for this test at our institution is typically around 2 weeks. Therefore, due to the lack of clinical usefulness, fecal calprotectin testing was not ordered in either of the two cases presented. C-reactive protein (CRP) testing was also not ordered in either case and is not routinely ordered in our clinic for CIC due to the nonspecific nature of elevated CRP values in advanced cancer patients, many of whom have chronic, systemic inflammation from their underlying malignant disease [34]. Unlike in cases of IBD, CRP levels have been found to be an unreliable surrogate for assessing treatment response or disease recurrence in CIC [35]. In future hypothesis-driven research, routine assessment of inflammatory markers in combination with diagnostic imaging could provide quantitative matrix measures of CIC improvement and further the understanding of the effects of increased vedolizumab dosing.

In conclusion, CIC is a common side effect of all currently approved ICIs and is frequently treated with vedolizumab. The standard 300-mg dose of vedolizumab may prove insufficient to resolve CIC in some patients, as was the case with the two patients presented here. Clinicians should consider that patient weight and hypoalbuminemia may impact vedolizumab trough levels and clinical response, necessitating reassessment of the dosing regimen of vedolizumab for clinically unresponsive CIC patients. Further prospective studies are warranted to clarify the role and benefit of dose doubling of vedolizumab in patients with CIC.

## Figures and Tables

**Table 1:** CBC values at the time of following clinical visits.

	**First dose of VDZ**	**600-mg dose of VDZ**	**Post 600-mg dose of VDZ** ^**a**^
*Case #1*			
RBC (10^6^/*μ*L)	4.36	4.36	3.95
Hemoglobin (g/dL)	11.9	11.5	10.4
Hematocrit (%)	34.80	35.00	32.30
MCV (fL)	79.8	80.4	81.8
RDW (%)	17.20	17.60	17.40
Platelets (K/*μ*L)	336	483	457
WBC (10^3^/*μ*L)	9.1	9.1	13.8
Lymphocytes (%)	7	16	11
Monocytes (%)	2	8	8
Eosinophils (%)	0	3	0
Basophils (%)	1	1	0
Neutrophils (%)	90	71	81
*Case #2*			
RBC (10^6^/*μ*L)	3.41	2.52	3.01
Hemoglobin (g/dL)	10.4	7.5	9.1
Hematocrit (%)	31.40	22.80	27.60
MCV (fL)	92	90.2	91.6
RDW (%)	15.90	16.80	18.60
Platelets (K/*μ*L)	384	259	691
WBC (10^3^/*μ*L)	14.5	10.2	10.8
Lymphocytes (%)	26	—	27
Monocytes (%)	9	—	6
Eosinophils (%)	1	—	1
Basophils (%)	0	—	1
Neutrophils (%)	64	—	65

Abbreviation: VDZ, vedolizumab.

^a^CBC was obtained 14 days post 600-mg dose for Case #1 and 1-month post 600-mg dose of Case #2.

## Data Availability

The anonymized data generated in the present study may be requested from the corresponding author.

## Nomenclature

ICIs: immune checkpoint inhibitors
CIC: checkpoint inhibitor colitis
CTLA-4: cytotoxic T lymphocyte–associated protein 4
PD-1: programmed death protein 1
PD-L1: programmed death-ligand 1
IBD: inflammatory bowel disease
MAdCAM-1: mucosal addressin cellular adhesion molecule
